# Life satisfaction in emerging adulthood: a longitudinal study of social support, resilience, and gender differences

**DOI:** 10.3389/fpsyg.2025.1602140

**Published:** 2025-07-25

**Authors:** Egemen Hanımoğlu

**Affiliations:** Department of Psychological Counseling and Guidance, Faculty of Education, University of Çukurova, Adana, Türkiye

**Keywords:** social support, life satisfaction, psychological resilience, gender differences, longitudinal study, structural equation modeling

## Abstract

**Background:**

Emerging adulthood (ages 18–29) is a critical developmental period characterized by significant psychological and social transitions. While social support and psychological resilience have been consistently associated with wellbeing, their longitudinal associations with life satisfaction remain underexplored. Moreover, the potential moderating role of gender in these relationships is underrepresented in the existing literature, highlighting a gap this study seeks to address.

**Objectives:**

This study examines the longitudinal associations between social support and life satisfaction, investigates whether psychological resilience accounts for these associations, and explores the potential moderating role of gender over time. By employing a multi-wave design, it seeks to provide a nuanced understanding of how these psychological resources are interrelated across time.

**Methods:**

A total of 566 emerging adults (50.7% female, 49.3% male) participated in this three-wave longitudinal study conducted over 1 year with six-month intervals (T1, T2, T3). Structural Equation Modeling (SEM) was used to test direct and indirect effects of social support on life satisfaction through psychological resilience. Multi-group SEM and chi-square difference tests were conducted to assess gender differences in the proposed pathways.

**Results:**

Higher levels of social support at T1 were significantly associated with greater life satisfaction at T2 and T3 (*β* = 0.22, *p* < 0.01). Psychological resilience was found to partially account for this association (indirect effect: *β* = 0.25, *p* < 0.001), suggesting that resilience may function as a linking factor between social support and subsequent wellbeing. Gender appeared to moderate these associations (Δ*χ*^2^ = 16.27, *p* < 0.001), with stronger paths observed among women—particularly in the association between social support and resilience.

**Conclusion:**

This study contributes longitudinal evidence indicating that social support is positively associated with life satisfaction, both directly and through its associations with psychological resilience. The gender-specific patterns observed in the findings underscore the relevance of developing mental health interventions that consider individual differences in support utilization and coping strategies. These insights may inform theoretical frameworks and guide practical efforts to enhance wellbeing across diverse populations. Future research is encouraged to examine these associations across varying cultural and demographic contexts to further refine theoretical models and improve intervention strategies.

## 1 Introduction

Emerging adulthood (ages 18–29) is a transformative life stage marked by significant psychological, cognitive, and social transitions. This period encompasses major life changes, including higher education, workforce entry, financial independence, and romantic relationships, all of which influence psychological adaptation and life satisfaction (Arnett, 2000; Fry and Debats, 2010). While these transitions offer opportunities for personal growth, they also introduce stressors that may lead to fluctuations in wellbeing (Galambos et al., 2020; Schulenberg et al., 2004). Given these challenges, identifying the factors that sustain life satisfaction during this phase is particularly relevant. Life satisfaction, as a psychological construct, does not develop in isolation but is shaped by both personal attributes and external influences.

This study is grounded in three complementary psychological theories that illuminate the complex interplay between social support, resilience, and life satisfaction in emerging adulthood. Self-Determination Theory (SDT) posits that individuals experience optimal wellbeing when their basic psychological needs—autonomy, competence, and relatedness—are fulfilled (Deci and Ryan, 2000). These needs are deeply embedded in social contexts. For instance, perceived social support enhances relatedness and fosters a sense of competence during identity exploration and role transitions, making it a key predictor of wellbeing in emerging adults (Gaine et al., 2021). Longitudinal studies further suggest that autonomy-supportive environments can buffer the negative effects of stress and promote adaptive functioning across developmental stages (Stirnberg et al., 2024).

Stress and Coping Theory (Lazarus and Folkman, 1984) provides a complementary perspective by focusing on how individuals evaluate and respond to stressors. In this framework, psychological resilience functions as a dynamic process through which individuals adapt to adverse circumstances, often facilitated by social support (Matud et al., 2023; Park et al., 2024). Rather than viewing stress solely as a risk factor, this theory emphasizes the protective role of external and internal resources in maintaining life satisfaction over time.

Attachment Theory (Bowlby, 1988) further enriches the framework by emphasizing the emotional bonds formed in early life, which shape subsequent interpersonal relationships and stress responses. Secure attachment styles are associated with greater perceived support, more effective coping strategies, and enhanced psychological resilience, all of which contribute to higher life satisfaction (Germani et al., 2020; Peleg and Peleg, 2024). These three frameworks converge to suggest that the experience of support, the ability to adapt to stress, and the quality of interpersonal bonds are central to understanding variations in wellbeing during emerging adulthood.

The links between social support, psychological resilience, and life satisfaction are well-established in psychological literature, yet their long-term developmental interplay continues to receive growing scholarly attention. Foundational research has consistently demonstrated that social support functions as a critical protective factor in mitigating the adverse effects of stress on wellbeing (Cohen and Wills, 1985; Uchino, 2006). Particularly during periods of transition, perceived emotional and instrumental support fosters a sense of stability and belonging, which in turn promotes sustained life satisfaction (Lakey and Orehek, 2011). In parallel, psychological resilience—defined as the capacity to recover from adversity—has emerged as a core predictor of psychological wellbeing. The widely used Connor-Davidson Resilience Scale (CD-RISC) (Connor and Davidson, 2003) has helped establish resilience as a measurable, modifiable construct, associated with improved coping, self-regulation, and long-term mental health outcomes. Moreover, Bonanno (2004) emphasized the natural capacity for resilience as more common than previously believed, proposing that it serves not merely as a trait but as a dynamic process influenced by context and support systems.

Gender has been consistently shown to moderate the influence of both social support and resilience on life satisfaction, yet the underlying mechanisms are often under-theorized—particularly in non-Western contexts. Biopsychosocial models suggest that women are more likely to utilize emotion-focused coping strategies, seek interpersonal closeness, and respond to support through oxytocin-mediated bonding mechanisms (Matud et al., 2023; Taylor et al., 2000). In contrast, men are typically socialized into problem-focused coping, emphasizing autonomy and instrumental action, which may reduce their perceived benefit from emotional support (Lee et al., 2020). These patterns are further shaped by cultural expectations. In collectivistic societies such as Turkey, gender norms play a substantial role in defining acceptable coping behaviors. Women are often encouraged to express vulnerability and rely on familial support, while men may be expected to demonstrate stoicism and independence, even in the face of adversity (Peleg and Peleg, 2024). Despite these patterns, empirical evidence on gendered resilience and support processes in emerging adulthood—particularly within Middle Eastern or collectivist cultures—remains limited.

Building upon the identified research gaps and grounded in the theoretical frameworks discussed, this study systematically examines how social support and resilience influence life satisfaction over time. Accordingly, the following hypotheses are proposed:

*H1*: Higher levels of social support predict longitudinal increases in life satisfaction.

Although the direct effects of social support on life satisfaction are well documented, the developmental trajectory of this association remains underexplored. Social support provides emotional, informational, and instrumental resources that enhance wellbeing and buffer against stress (Cohen and Wills, 1985; Lakey and Orehek, 2011). In emerging adulthood—a period marked by relational instability and identity exploration—such support may be particularly influential. Therefore, we hypothesize that individuals with higher perceived social support will report increased life satisfaction over time.

*H2*: Psychological resilience mediates the relationship between social support and life satisfaction.

The underlying mechanisms through which social support enhances wellbeing merit further examination. Psychological resilience has been identified as a key mediator, enabling individuals to convert external support into internal coping capacity (Luthar and Cicchetti, 2001; Masten, 2013). Empirical studies have shown that social support facilitates resilience by promoting emotion regulation, adaptive coping, and self-efficacy (Clarke, 2024; Yıldırım and Tanrıverdi, 2020). Thus, we propose that resilience functions as a pathway through which social support leads to greater life satisfaction.

*H3*: Gender moderates the associations between social support, psychological resilience, and life satisfaction.

Prior research suggests that the benefits of social support and resilience may vary by gender. Women are more likely to engage in emotionally expressive coping and to rely on interpersonal support, while men often adopt problem-focused strategies and may underutilize emotional support (Matud et al., 2023; Taylor et al., 2000; Zhang et al., 2014). In collectivist cultures like Turkey, these gendered patterns are further reinforced by social expectations regarding independence, emotional restraint, and help-seeking (Peleg and Peleg, 2024). These differences suggest that gender may influence the strength or direction of the associations between support, resilience, and wellbeing. Accordingly, we hypothesize a moderating effect of gender.

By adopting a gender-sensitive and longitudinal approach, this study aims to provide new insights into how social and psychological resources shape life satisfaction trajectories. The findings may contribute to the development of culturally responsive mental health interventions designed to enhance wellbeing in emerging adults.

## 2 Methods

### 2.1 Research design

This study employed a longitudinal research design to examine the effects of social support and psychological resilience on life satisfaction. Data were collected at three time points (T1, T2, and T3) at six-month intervals, a timeframe informed by prior longitudinal studies on psychological wellbeing (e.g., Lucas, 2007; Voelkle and Oud, 2012), which suggest it effectively captures meaningful changes while minimizing participant burden. To assess individual changes over time, panel data analysis and Latent Growth Curve Modeling (LGM) were applied. Additionally, Structural Equation Modeling (SEM) with bootstrapping was conducted to test the mediating role of psychological resilience, while Multi-Group SEM and the chi-square difference test (Δ*χ*^2^ Test) evaluated the moderating effect of gender.

In this study, the traditional Cross-Lagged Panel Model (CLPM) was employed to examine the directional associations among social support, psychological resilience, and life satisfaction over a period of three waves. Although CLPM is extensively utilized in longitudinal research, it possesses certain limitations, notably its incapacity to differentiate between within-person and between-person effects, which may result in the conflation of estimates of change over time (Hamaker, 2023; Shi et al., 2022). Alternative models, such as the Random Intercept Cross-Lagged Panel Model (RI-CLPM), address this issue by separating time- invariant between-person variance, thus isolating within-person fluctuations (Burns et al., 2020; Orth et al., 2021). However, RI-CLPM necessitates larger sample sizes, intricate modeling assumptions, and a pronounced theoretical emphasis on intraindividual variability (Lüdtke and Robitzsch, 2022). The present study’s primary focus was on general directional trends as opposed to person-specific processes; moreover, the three-wave design utilized, with six-month intervals, proved to be more compatible with the traditional CLPM framework (Lucas, 2023). Consequently, CLPM was selected as the most suitable model to address the research questions, while its limitations are acknowledged and discussed in the final section of the paper.

### 2.2 Participants

The sample size was determined using G*Power 3.1 software (Faul et al., 2007). Based on a medium effect size (f^2^ = 0.15), a significance level (*α* = 0.05), and a statistical power of 95% (1 − *β* = 0.95), a minimum of 138 participants was required. However, to enhance the generalizability of the findings, a larger sample of 566 participants was recruited.

Table 1 presents the demographic characteristics of the sample across the three time points. The first data collection (T1) was conducted at the beginning of the study, measuring participants’ social support, psychological resilience, and life satisfaction. The second measurement (T2) took place 6 months after T1, reassessing these variables. Finally, the T3 measurement was conducted 12 months after the study began, with all scales administered one last time. While 566 participants completed the T1, 482 remained in T2 (15% attrition), and 410 participated in T3 (27.5% total attrition).

**Table 1:** Demographic characteristics of the sample (*N* = 566 at T1, *N* = 482 at T2, *N* = 410 at T3).

Variable	T1 (*N* = 566)	T2 (*N* = 482)	T3 (*N* = 410)
Gender			
Female	287 (50.7%)	240 (49.8%)	200 (48.8%)
Male	279 (49.3%)	242 (50.2%)	210 (51.2%)
Education level			
Undergraduate	440 (77.7%)	375 (77.8%)	310 (75.6%)
Graduate	126 (22.3%)	107 (22.2%)	100 (24.4%)

The gender distribution at T1 was balanced, with 50.7% female (*n* = 287) and 49.3% male (*n* = 279) participants. However, in the follow-up measurements, the proportion of female participants decreased to 48.8% at T3, while the proportion of male participants increased to 51.2%.

Regarding education level, the majority of participants were undergraduate students (77.7%, *n* = 440), while 22.3% (*n* = 126) were graduate students. Changes in educational levels over time were monitored, with the proportion of graduate students increasing to 24.4% at T3.

In summary, Table 1 illustrates participant attrition over time and changes in gender and education levels, reflecting the longitudinal nature of the study.

### 2.3 Data collection instruments

#### 2.3.1 Satisfaction with Life Scale (SWLS)

Developed by Diener et al. (2010) and adapted into Turkish by Durak et al. (2010), the Satisfaction with Life Scale (SWLS) consists of five items. Participants rate each item on a 7-point Likert scale ranging from “1 = Strongly Disagree” to “7 = Strongly Agree.” Higher SWLS scores indicate greater life satisfaction. In this study, the internal consistency reliability coefficients of the scale were calculated as 0.85 at T1, 0.86 at T2, and 0.87 at T3.

#### 2.3.2 Multidimensional Scale of Perceived Social Support (MSPSS)

Developed by Zimet et al. (1988) and validated in Turkish by Eker et al. (2001), the MSPSS assesses individuals’ perceived adequacy of social support from three different sources: family (items 3, 4, 8, 11), friends (items 6, 7, 9, 12), and a significant other (items 1, 2, 5, 10). The scale consists of 12 items and uses a 7-point Likert scale.

The subscale scores are summed to obtain the total scale score. Subscale scores range from 4 to 28, while the total scale score ranges from 7 to 84. Higher scores indicate a higher level of perceived social support. The Cronbach’s alpha coefficients for the subscales were found to range between 0.80 and 0.85 (Eker et al., 2001). In this study, the internal consistency reliability coefficients of the scale were found to be 0.88 at T1, 0.89 at T2, and 0.88 at T3.

#### 2.3.3 Brief Resilience Scale (BRS)

The Brief Resilience Scale (BRS) was developed by Smith et al. (2008) and adapted into Turkish by Doğan (2015), who examined its psychometric properties. The factor loadings of the scale items range from 0.63 to 0.79, while the item-total correlation values range from 0.49 to 0.66.

The scale consists of six items and is rated on a 5-point Likert scale: “1 = Not at all true,” “2 = Somewhat untrue,” “3 = Neutral,” “4 = Somewhat true,” and “5 = Completely true.” Items 2, 4, and 6 are reverse-coded.

Higher scores on the scale indicate greater psychological resilience. The Cronbach’s alpha reliability coefficient for the scale was found to be 0.83. In this study, the reliability coefficients were calculated as 0.80 at T1, 0.82 at T2, and 0.81 at T3.

#### 2.3.4 Demographic information form

A demographic form was used to collect participant information such as age, gender, and educational background. This form was designed by the researcher.

### 2.4 Data collection

The first data collection process was conducted in September 2023 (T1), the second in March 2024 (T2), and the third in September 2024 (T3). Each data collection phase was carried out at six-month intervals to track changes in social support, psychological resilience, and life satisfaction over time. These intervals were chosen to minimize potential psychological effects related to academic terms and life transitions.

Individuals who agreed to participate in the study received an online survey link via email and social media platforms. To ensure consistency in measurements, the same set of surveys was administered at each time point. Participants were asked to complete the Satisfaction with Life Scale (SWLS), the Multidimensional Scale of Perceived Social Support (MSPSS), and the Brief Resilience Scale (BRS).

Each participant completed the survey individually in approximately 20 min. To mitigate sample attrition, reminder emails were sent before T2 and T3, and small incentives were provided. Over time, some participants dropped out of the study, with 566 participants at T1, 482 at T2, and 410 at T3, indicating a 27.5% sample attrition rate over 1 year.

All data were collected anonymously, and participants were assured that their responses would remain confidential. The study was approved by the Çukurova University Ethics Committee, and the data collection process was conducted in accordance with ethical guidelines.

### 2.5 Missing data and attrition analysis

Participant attrition is a common concern in longitudinal research, as it may compromise the internal validity of findings (Little and Rubin, 2002). In the present study, 566 participants completed the Time 1 (T1) assessment, 482 completed Time 2 (T2), and 410 completed Time 3 (T3), yielding an overall retention rate of approximately 72.5% from T1 to T3.

To evaluate potential systematic attrition bias, independent samples *t*-tests were conducted to compare participants who completed all three waves (*n* = 410) with those who dropped out after T1 or T2 (*n* = 156) on key study variables. No significant differences were found for social support, *t* (564) = 1.12, *p* = 0.26, or life satisfaction, *t* (564) = 0.87, *p* = 0.39, suggesting that attrition was random rather than systematic.

Additionally, Little’s MCAR test was conducted to examine the missing data mechanism. The test yielded a non-significant result, *χ*^2^ (26) = 23.47, *p* = 0.61, supporting the assumption that data were missing completely at random (MCAR) (Schafer and Graham, 2002).

To handle missing data, Full Information Maximum Likelihood (FIML) estimation was employed. FIML provides unbiased parameter estimates under the assumptions of MCAR or MAR and is widely recognized as a robust approach in longitudinal designs (Enders, 2010).

Further comparisons were made to examine potential attrition bias related to demographic and psychological variables. An independent samples *t*-test indicated no significant difference in baseline resilience scores between completers and dropouts, *t* (498) = 0.72, *p* = 0.47. Similarly, a chi-square test showed no significant association between gender and attrition status, *χ*^2^ (1, *N* = 500) = 1.39, *p* = 0.24. These results further support the assumption that attrition did not systematically bias the data.

## 3 Results

Before testing the hypotheses, preliminary analyses were conducted to ensure that the fundamental assumptions of the statistical methods were met. Normality was assessed using the Shapiro–Wilk test, along with skewness and kurtosis values, for all variables across T1, T2, and T3 measurements. In all cases, *p*-values exceeded 0.05, indicating that the normality assumption was satisfied. Additionally, skewness and kurtosis values ranged between −1 and +1, confirming a normal distribution (Tabachnick and Fidell, 2019).

Multicollinearity, a critical assumption in regression-based analyses, was examined through Variance Inflation Factor (VIF) values. The VIF for social support was 2.1, and for psychological resilience, 2.35. While VIF values below 10 are generally acceptable, values under 5 provide more reliable results, suggesting that multicollinearity was not a concern (Hair et al., 2018). Since life satisfaction was the dependent variable, its VIF was not calculated.

To assess the independence of observations, the Durbin-Watson test was performed to check for autocorrelation. The results were 1.82 for social support and 1.95 for life satisfaction, both falling within the acceptable range of 1.5–2.5, confirming the absence of autocorrelation (Field, 2018; Gujarati and Porter, 2009). The Durbin-Watson test was not computed for psychological resilience as it was not an independent variable.

The reliability of the measurement instruments was evaluated using Cronbach’s Alpha. The coefficients were.89 for social support, 0.87 for psychological resilience, and.85 for life satisfaction, indicating high internal consistency (Nunnally and Bernstein, 1994). Similar results were found across T1, T2, and T3, further supporting the reliability of the scales.

After verifying assumptions and reliability, descriptive statistics and correlation analyses were conducted to examine relationships among the study variables. Table 2 presents the mean (M), standard deviation (SD), and correlation coefficients across time points.

**Table 2:** Descriptive statistics and correlations of variables (T1, T2, T3).

Variable	M (T1)	SD (T1)	M (T2)	SD (T2)	M (T3)	SD (T3)	1	2	3
1. Social support	3.85	0.72	3.92	0.70	3.97	0.68	1	0.52**	0.45**
2. Psychological resilience	3.62	0.68	3.78	0.65	3.85	0.63	0.52**	1	0.56**
3. Life satisfaction	3.74	0.75	3.81	0.72	3.89	0.70	0.45**	0.56**	1

**All correlations are significant at *p* < 0.01.

The correlation results indicate significant relationships between social support, psychological resilience, and life satisfaction, aligning with theoretical expectations. Social support was positively correlated with psychological resilience (r = 0.52, *p* < 0.01) and life satisfaction (r = 0.45, *p* < 0.01). Additionally, a strong positive association was found between psychological resilience and life satisfaction (r = 0.56, *p* < 0.01). These findings suggest that both social support and psychological resilience may be linked to life satisfaction, providing empirical support for subsequent hypothesis testing (Tabachnick and Fidell, 2019; Hair et al., 2018).

### 3.1 The relationship between social support and life satisfaction (hypothesis 1)

In this study, a Cross-Lagged Panel Model (CLPM) was used to examine the bidirectional relationship between social support and life satisfaction over time. The model fit indices were calculated as CFI = 0.97, TLI = 0.96, RMSEA = 0.05, and SRMR = 0.04, indicating a good model fit (Hu and Bentler, 2009). As shown in Table 3, social support at T1 had a positive and significant association with life satisfaction at T2 (*β* = 0.22, *p* < 0.01), reflecting a moderate association strength (Kline, 2015). This suggests that individuals with greater perceived support tend to report noticeably higher levels of life satisfaction over time. Similarly, social support at T2 positively predicted life satisfaction at T3 (*β* = 0.20, *p* < 0.01), also reflecting a moderate and practically meaningful association. However, the cross-lagged associations of life satisfaction on social support were not found to be significant (T1 → T2: *β* = −0.06, *p* = 0.21; T2 → T3: *β* = −0.05, *p* = 0.27). These results suggest that social support may be a predictor of future life satisfaction, but life satisfaction is not significantly associated with subsequent social support.

**Table 3:** Standardized regression coefficients for cross-lagged panel model.

Pathway	*β*	SE	*p*-value	R^2^
Social support (T1) → Life satisfaction (T2)	0.22	0.07	< 0.01	0.18
Social support (T2) → Life satisfaction (T3)	0.20	0.06	< 0.01	0.16
Life satisfaction (T1) → Social support (T2)	−0.06	0.05	0.21	0.05
Life satisfaction (T2) → Social support (T3)	−0.05	0.04	0.27	0.04
Social support (T1) → Social support (T2)	0.52	0.06	< 0.001	0.50
Social support (T2) → Social support (T3)	0.49	0.05	< 0.001	0.48
Life satisfaction (T1) → Life satisfaction (T2)	0.48	0.06	< 0.001	0.47
Life satisfaction (T2) → Life satisfaction (T3)	0.46	0.05	< 0.001	0.45

Model Fit Indices: CFI = 0.97, TLI = 0.96, RMSEA = 0.05, SRMR = 0.04.

Additionally, there was a strong relationship between social support at T1 and T2 (*β* = 0.52, *p* < 0.001). A similar significant association was found between social support at T2 and T3 (*β* = 0.49, *p* < 0.001). The same pattern was observed for life satisfaction, with a significant relationship between T1 and T2 (*β* = 0.48, *p* < 0.001) and between T2 and T3 (*β* = 0.46, *p* < 0.001).

These findings indicate that individuals exhibit relatively stable tendencies over time in terms of both social support and life satisfaction. In other words, previous levels of social support and life satisfaction were predictive of their future values.

**Figure 1:**
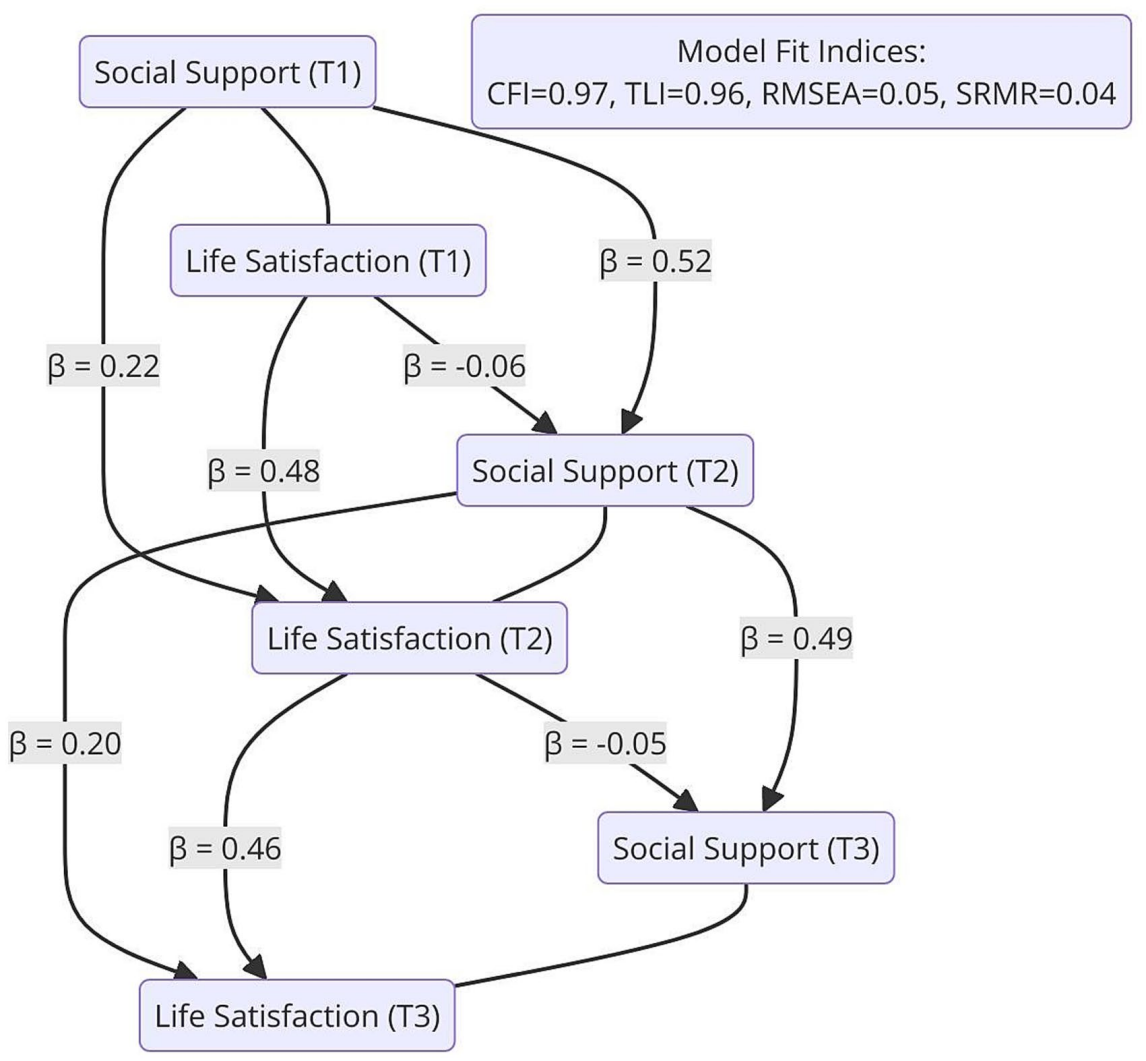
Cross-lagged panel model (CLPM) path diagram. Cross-lagged panel model illustrating the longitudinal associations between social support and life satisfaction across three time points (T1–T3). Standardized beta coefficients are shown along the paths. Model fit indices: CFI = 0.97, TLI = 0.96, RMSEA = 0.05, SRMR = 0.04.

Overall, the model results indicate that social support was significantly associated with higher levels of life satisfaction over time, whereas the association between life satisfaction and subsequent social support was not statistically significant. All relationships and regression coefficients are visualized in Figure 1, which presents the Cross-Lagged Panel Model (CLPM) Path Diagram.

Figure 1 summarizes the cross-lagged panel model (CLPM), which illustrates the time-dependent relationships between social support and life satisfaction. The arrows in the diagram represent the directional associations between variables, covering both direct and cross-lagged paths at T1 (first measurement), T2 (second measurement), and T3 (third measurement).

The diagram also includes standardized regression coefficients. The associations from social support to life satisfaction are positive and significant, whereas the reverse paths (life satisfaction → social support) are not significant. Additionally, both social support and life satisfaction show continuity over time. The model fit indices were calculated as CFI = 0.97, TLI = 0.96, RMSEA = 0.05, and SRMR = 0.04, indicating a good model fit.

In cross-lagged panel models (CLPM), sample size is a crucial factor influencing the model’s predictive power. Within the structural equation modeling (SEM) framework, determining whether small cross-lagged associations are statistically significant is an important methodological consideration (Cohen, 1988; Kline, 2015).

In this study, T1 included 566 participants, T2 had 482 participants, and T3 had 410 participants. The power analysis conducted based on the sample size and parameter estimations indicates that the social support → life satisfaction pathways had sufficient statistical power. Specifically, the significant results for *β* = 0.22 and *β* = 0.20 suggest that the model is adequately powered to detect these associations.

However, for the life satisfaction → social support pathways, the coefficients were small (*β* = −0.06, *β* = −0.05) and not statistically significant (*p* > 0.05), suggesting that the statistical power for these paths may be lower (Cohen, 1988). This finding aligns with previous research indicating that the statistical utility of cross-lagged pathways in CLPM can vary depending on the sample size (Little, 2013).

In longitudinal studies, sample attrition is a critical factor that can impact internal validity (Little and Rubin, 2002). In this study, 566 participants completed T1, while 482 participants remained at T2, and 410 participants completed T3. To assess whether attrition was systematic, participants who dropped out after T1 were compared with those who remained in the study.

Results from *t*-tests (*p* > 0.05) indicate that there were no significant differences in social support and life satisfaction between the dropout group and the retained participants. This suggests that the missing data followed a Missing Completely at Random (MCAR) pattern (Schafer and Graham, 2002), meaning that attrition was unlikely to introduce bias into the study findings.

In addition, the Full Information Maximum Likelihood (FIML) method was used in the analyses. FIML is a widely recommended approach for minimizing the impact of missing data (Enders, 2010). As a result, it was assessed that missing data did not significantly distort the overall model outcomes.

While the Cross-Lagged Panel Model (CLPM) provides insights into temporal relationships between social support and life satisfaction, it does not fully account for individual variability. In contrast, the Latent Growth Model (LGM) offers a more suitable framework for examining overall change trends over time and evaluating initial levels and change rates among individuals.

To examine the longitudinal changes in life satisfaction, a Latent Growth Model (LGM) was applied. The model assessed life satisfaction at three time points, analyzing both the initial level (Intercept) and the trend of change over time (Slope).

As shown in Table 4, the estimated mean initial level of life satisfaction was 3.74 (SE = 0.05, *p* < 0.001), indicating that participants generally started with a high level of life satisfaction.

**Table 4:** Estimated parameter values, standard errors, and significance levels.

Parameter	Estimate	Standard error	*p*-value
Intercept mean (initial level)	3.74	0.05	< 0.001
Slope mean (rate of change)	0.09	0.02	< 0.01
Intercept variance (initial level variance)	0.42	0.07	< 0.001
Slope variance (change rate variance)	0.06	0.02	< 0.05
Intercept-slope covariance	0.10	0.04	< 0.05

Additionally, the slope coefficient was positive and significant (*β* = 0.09, SE = 0.02, *p* < 0.01), suggesting a small but significant upward trend in life satisfaction over time.

To understand individual differences within the model, the variance of the initial level (intercept) and the rate of change (slope) was examined. The variance of the initial level was found to be 0.42 (SE = 0.07, *p* < 0.001), while the variance of the rate of change was 0.06 (SE = 0.02, *p* < 0.05). This finding suggests that there are significant individual differences in both the starting levels of life satisfaction and the rate of change over time.

Additionally, the covariance between the initial level and the slope was 0.10 (SE = 0.04, *p* < 0.05), indicating that individuals who started with higher life satisfaction tended to show less increase over time.

To evaluate how well the applied model fit the data, various fit indices were examined. The CFI value was 0.96, and the TLI value was 0.95, both exceeding the 0.95 threshold, supporting a good model fit. Furthermore, the RMSEA value was 0.04, and the SRMR value was 0.03, both below 0.05, indicating a strong alignment between the model and the data. Overall, these fit indices suggest that the model provides an acceptable level of fit and is consistent with observed changes in life satisfaction over time.

To determine which model best explains the changes in life satisfaction, linear, freely estimated slope, and quadratic growth models were compared.

As shown in Table 5, the freely estimated slope model (AIC = 10528.93, BIC = 10584.21) had the lowest information criteria values, indicating the best fit. However, the difference between the freely estimated slope model and the linear and quadratic models was not statistically significant (*p* > 0.05).

**Table 5:** Fit comparisons of linear, freely estimated slope, and quadratic models.

Model	AIC	BIC	Δ*χ*^2^ (comparative fit)	*p*-value
Linear model (linear growth)	10532.45	10589.67	–	–
Freely estimated slope model	10528.93	10584.21	3.52	0.061
Quadratic model (quadratic growth)	10530.72	10586.89	1.73	0.189

This suggests that the overall pattern of change is largely linear, but individual differences in the rate of change exist.

**Figure 2:**
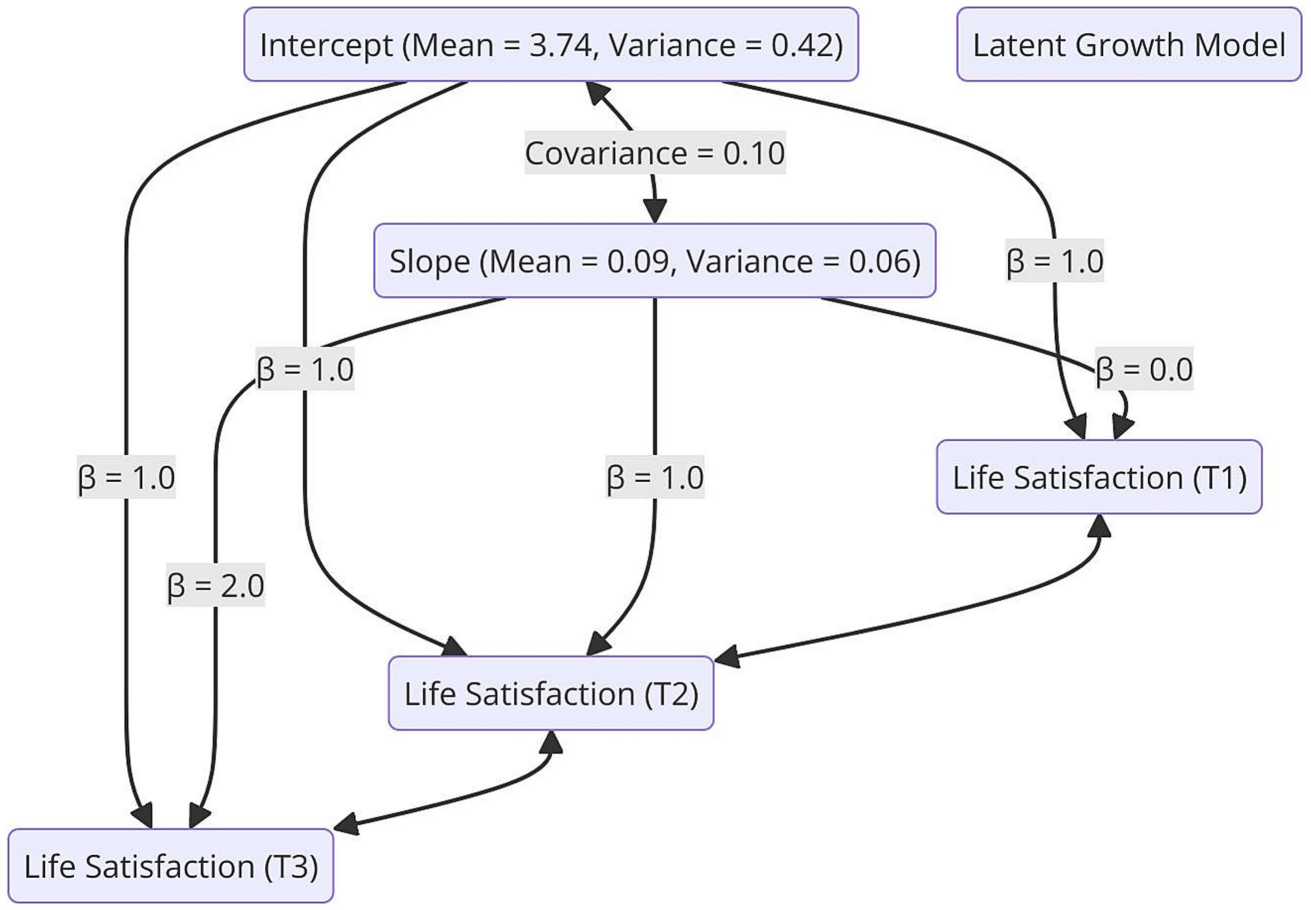
Latent growth model (LGM) path diagram. The intercept and slope factors are specified, including their means, variances, and covariance. Standardized path coefficients (*β*) indicate the contribution of each latent factor to the observed variables across time.

To visualize the results of the Latent Growth Model (LGM), two different graphs were created. Figure 2 presents the LGM path diagram, illustrating the relationships between the initial level, the slope factor, and time points. The diagram highlights that all time points were significantly influenced by the initial level, while the slope factor reflected changes over time.

**Figure 3:**
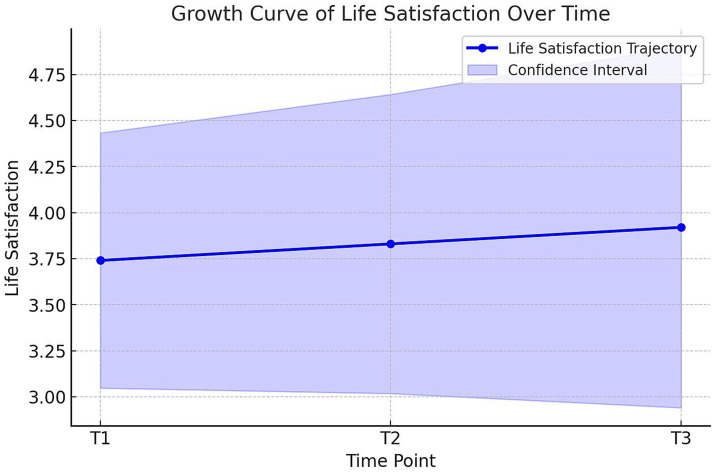
Trajectory of life satisfaction over time. The solid blue line represents the trajectory of mean life satisfaction scores from T1 to T3, and the shaded area represents the confidence interval.

Furthermore, the change trajectory over time (Figure 3) demonstrates a general positive increase in individuals’ life satisfaction, while the width of the confidence intervals reflects individual differences.

Overall, the LGM findings indicate that individuals’ life satisfaction increases over time and that there are individual differences in this change. The good model fit suggests that the structure used to explain this change is statistically valid. The fact that the freely estimated slope model provides the best fit implies that the rate of change in life satisfaction varies among individuals. Finally, the negative covariance between the initial level and the slope suggests that individuals with higher initial life satisfaction tend to show less change over time.

### 3.2 The mediating role of psychological resilience in the relationship between social support and life satisfaction (hypothesis 2)

In this study, structural equation modeling (SEM) was used to test the mediating role of psychological resilience in the association between social support and life satisfaction. The model was estimated using the maximum likelihood (ML) method, and the fit indices indicated a good model fit (CFI = 0.96, TLI = 0.94, RMSEA = 0.04, SRMR = 0.03). These values meet the thresholds recommended by Hu and Bentler (2009) (CFI and TLI ≥ 0.95, RMSEA ≤ 0.06, SRMR ≤ 0.08), supporting the model’s adequacy.

When examining the model results summarized in Table 6, it was found that social support (T1) was strongly associated with psychological resilience (T2) (*β* = 0.51, *p* < 0.001), indicating a large effect size, and suggesting that higher levels of social support are linked to greater resilience. Psychological resilience (T2) significantly predicted life satisfaction (T3) (*β* = 0.48, *p* < 0.001), also reflecting a strong practical association, emphasizing resilience as a robust predictor of future wellbeing. Moreover, social support (T1) also had a direct and significant association with life satisfaction (T3) (*β* = 0.22, *p* < 0.001), indicating a partial mediation role of psychological resilience.

**Table 6:** Structural equation model (SEM) regression results.

Pathway	*β*	SE	*p*	95% confidence interval
Social support (T1) → Psychological resilience (T2)	0.51	0.07	<0.001	[0.37, 0.65]
Psychological resilience (T2) → Life satisfaction (T3)	0.48	0.06	<0.001	[0.36, 0.60]
Social support (T1) → Life satisfaction (T3) (Direct Effect)	0.22	0.05	<0.001	[0.12, 0.32]
Social support (T1) → Psychological resilience (T2) → Life Satisfaction (T3) (Indirect Effect)	0.25	0.04	<0.001	[0.18, 0.32]

To determine whether the indirect pathway was statistically significant, the Bootstrap method (95% confidence intervals, 5,000 resamples) was applied. The results supported that the social support (T1) → psychological resilience (T2) → life satisfaction (T3) pathway showed a significant indirect association (*β* = 0.25, 95% CI [0.18, 0.32], *p* < 0.001). The indirect association of social support with life satisfaction through psychological resilience (*β* = 0.25) indicates a moderate-to-strong mediated association, which is both statistically and practically meaningful.

Additionally, the Sobel test also confirmed the significance of this indirect association (z = 4.89, *p* < 0.001). These findings indicate that psychological resilience (T2) partially mediates the association between social support (T1) and life satisfaction (T3).

**Figure 4:**
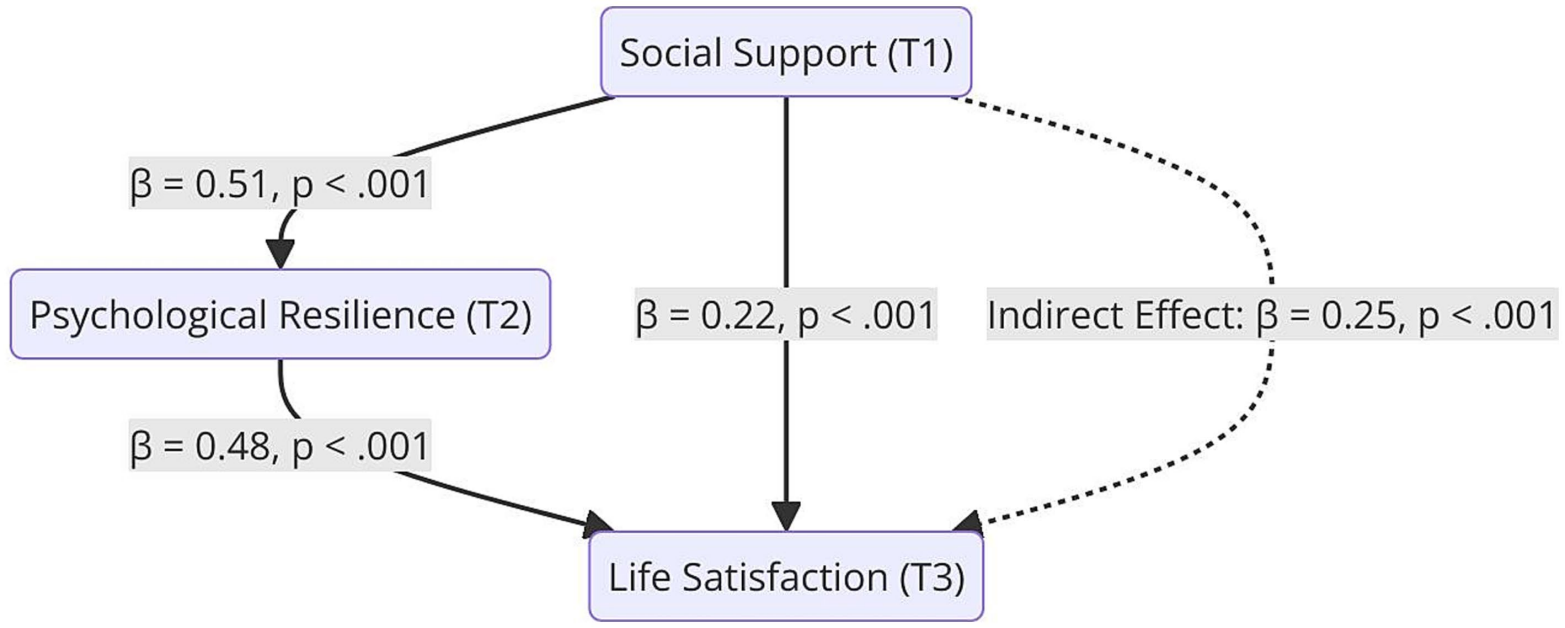
The mediating role of psychological resilience: social support and life satisfaction model. Mediation model illustrating the association between social support at T1 and life satisfaction at T3, with psychological resilience at T2 as a mediating variable. Standardized path coefficients (*β*) and significance levels are presented. The indirect association between social support and later life satisfaction via resilience was statistically significant.

Figure 4 illustrates the mediating role of psychological resilience (T2) in the association between social support (T1) and life satisfaction (T3). Solid lines represent direct pathways, the dashed line represents the indirect association through psychological resilience.

As shown in Figure 4, the predictive association between social support and life satisfaction appears to be strengthened through the mediation of psychological resilience. Additionally, all pathways are statistically significant (*p* < 0.001).

### 3.3 The moderating role of gender on social support and psychological resilience (hypothesis 3)

A multi-group structural equation model (Multi-group SEM) was used to test the moderating role of gender on the associations between social support, psychological resilience, and life satisfaction. Within this analysis, separate structural models were estimated for men and women, and the statistical significance of differences between these pathways was evaluated using the chi-square difference test (Δ*χ*^2^).

To test gender moderation, two models were created. In the constrained model, it was assumed that the paths were equal for men and women, whereas in the unconstrained model, these paths were allowed to vary. The difference between the two models was evaluated using the chi-square difference test. The results showed that the chi-square value of the unconstrained model was *χ*^2^ = 312.45 (df = 120, *p* < 0.001), while the chi-square value of the constrained model was *χ*^2^ = 328.72 (df = 122, *p* < 0.001). The chi-square difference test yielded Δ*χ*^2^ = 16.27, *p* < 0.001. This result indicates a significant difference between genders, suggesting that the association between social support and psychological resilience varies based on gender.

Fit indices were examined to evaluate the overall fit of the model. The model fit indices were within the acceptable recommended thresholds: CFI = 0.95, TLI = 0.94, RMSEA = 0.05, and SRMR = 0.04. These values indicate that the model is appropriate for testing the moderating role of gender (Hu and Bentler, 2009). Additionally, an alternative model comparison was conducted by examining Bayesian Information Criterion (BIC) values. The BIC value for the constrained model was 10,586.72, while the BIC value for the unconstrained model was 10,575.61. The lower BIC value for the unconstrained model supports the notion that gender is a significant moderator.

The structural paths estimated for men and women are presented in Table 7. The association of social support with psychological resilience was *β* = 0.55 (*p* < 0.001) for women, reflecting a strong predictive relationship, while it was *β* = 0.47 (*p* < 0.001) for men, indicating a moderate-to-strong association. These results suggest that women may benefit more from social support in building resilience, potentially due to gender-specific coping mechanisms. Similarly, the path from psychological resilience to life satisfaction was *β* = 0.50 for women and *β* = 0.42 for men, both statistically significant. These coefficients reflect large and moderate-to-large associations, respectively, and indicate that resilience is strongly linked to wellbeing, particularly for women.

**Table 7:** Structural path coefficients by gender.

Structural path	Men (*β*)	*p*	Women (*β*)	*p*	Δ*χ*^2^	*p* (difference)
Social support (T1) → Psychological resilience (T2)	0.47	<0.001	0.55	<0.001	7.89	< 0.01
Psychological resilience (T2) → Life satisfaction (T3)	0.42	<0.001	0.50	<0.001	5.23	< 0.05

These findings suggest that gender plays a moderating role in the associations between social support, psychological resilience, and life satisfaction. Specifically, the association between social support and psychological resilience was found to be stronger for women. Similarly, the association between psychological resilience and life satisfaction was also more pronounced among women. These results indicate that women benefit more from social support, enhancing their psychological resilience, which in turn is associated with higher life satisfaction.

**Figure 5:**
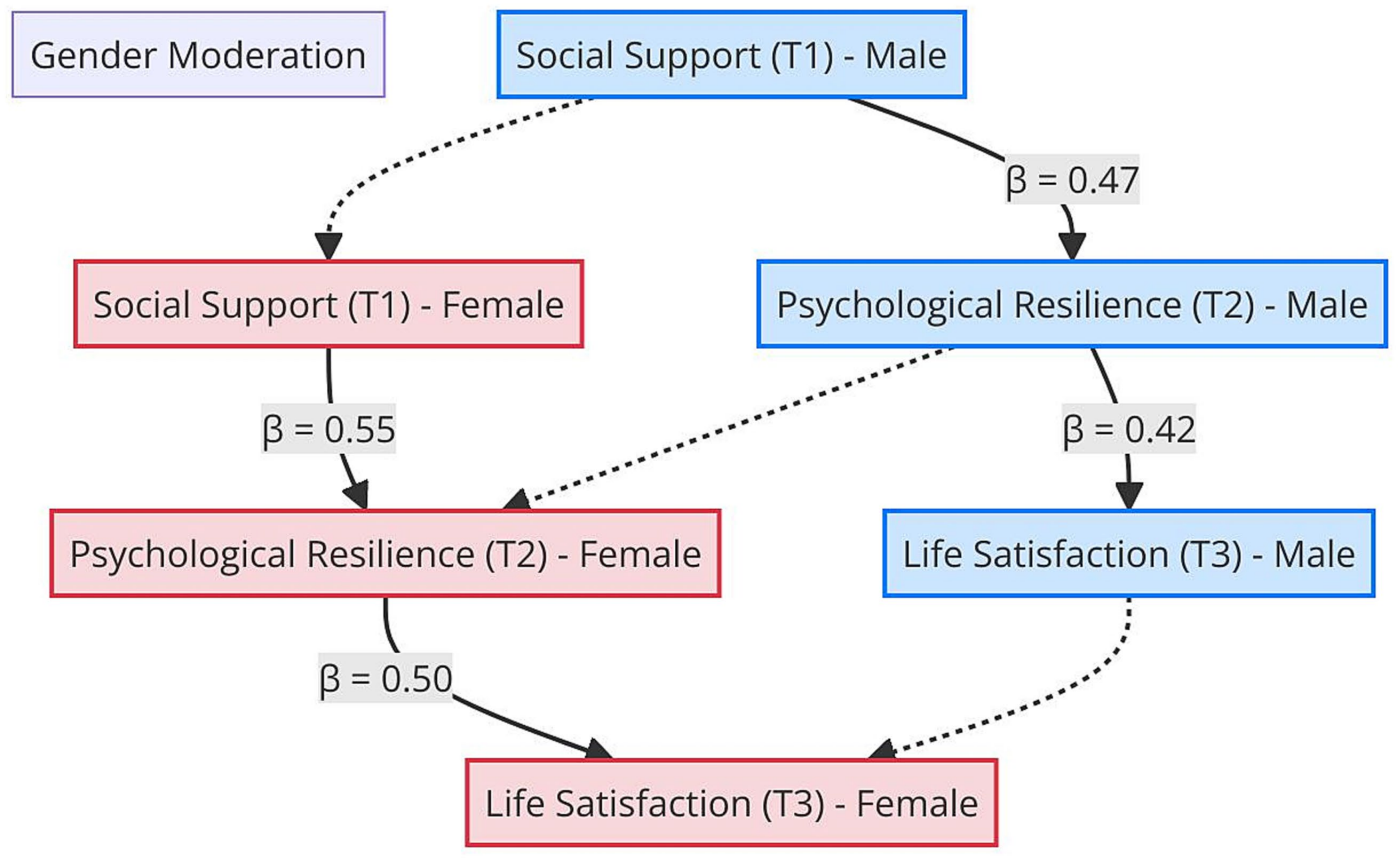
The model of social support and life satisfaction with gender moderation. Associations among social support, psychological resilience, and life satisfaction are presented separately for male and female participants. Standardized path coefficients (*β*) are displayed for each group, reflecting potential gender-based differences in the strength of these relationships.

The path diagram related to these findings is presented in Figure 5. In the figure, paths for women are shown in red, while paths for men are shown in blue. Dashed lines represent indirect pathways, highlighting how the paths from social support to psychological resilience and from psychological resilience to life satisfaction differ by gender.

In conclusion, the analyses indicate that gender moderates the association between social support and psychological resilience, with stronger associations observed for women. The statistical significance of the *χ*^2^ difference test, the model fit indices being within acceptable limits, and the BIC comparison favoring the unconstrained model all provide statistical support for the moderating role of gender.

## 4 Discussion

### 4.1 The relationship between social support and life satisfaction (hypothesis 1)

The findings of this study are consistent with the hypothesis that perceived social support is positively associated with life satisfaction over time. Results from the Cross-Lagged Panel Model (CLPM) indicated that higher levels of social support at earlier time points were significantly associated with subsequent levels of life satisfaction, whereas the reverse association was not statistically significant. These results highlight the temporal ordering of these constructs, suggesting that perceived social support may precede changes in life satisfaction during emerging adulthood.

This pattern aligns with previous longitudinal research (Luhmann et al., 2012; Chia et al., 2024), which has identified sustained associations between social support and life satisfaction across time. By employing a three-wave design, the present study contributes to a clearer understanding of temporal associations, offering stronger grounds for interpreting the directionality of relationships compared to cross-sectional designs. From a theoretical standpoint, these results are consistent with the stress-buffering model (Cohen and Wills, 1985), which frames social support as a resource that is associated with psychological wellbeing under conditions of stress. Additionally, frameworks such as attachment theory (Bowlby, 1969) and self-regulation theory (Baumeister and Vohs, 2007) may offer complementary insights into the role of sustained, supportive relationships in relation to wellbeing.

Interestingly, life satisfaction did not show a significant association with subsequent social support in the model. While social support was significantly associated with later life satisfaction, the reverse association—from life satisfaction to perceived social support—was not statistically significant. Several theoretical and methodological explanations may help account for this asymmetry. One possibility is the presence of ceiling effects, where individuals with already high levels of life satisfaction demonstrate limited variability across time, reducing the likelihood of observing predictive associations (Willroth et al., 2021). Cultural factors may also contribute; in collectivist societies such as Turkey, help-seeking behaviors are often influenced more by normative expectations than by subjective wellbeing (Tsai and Kimel, 2021; Krys et al., 2019). Moreover, personality traits or emotional stability may moderate these relationships over time, making them more complex than what is captured by global life satisfaction scales (Clark et al., 2020). These factors collectively suggest that positive internal states alone may not be sufficient to activate or sustain external social resources, especially in culturally or psychologically constrained contexts.

The observed stability of both social support and life satisfaction over time may reflect the trait-like nature of these constructs during emerging adulthood. Individuals reporting initially high levels of support and satisfaction tended to maintain these levels across the measurement points. This pattern suggests potential benefits of early enhancement of support systems, particularly within structured environments. Practical implications may include the design of peer support initiatives within educational or digital contexts, which could help reinforce perceptions of support and contribute to sustained psychological wellbeing.

### 4.2 The mediating role of psychological resilience (hypothesis 2)

Findings from this study suggest that psychological resilience may partially mediate the association between perceived social support and life satisfaction. Structural Equation Modeling results indicated that higher levels of social support at T1 were associated with increased resilience at T2, which in turn was linked to higher life satisfaction at T3. This potential mediational pathway was supported by both bootstrap analyses and the Sobel test.

These results are in line with previous research that identifies psychological resilience as a plausible pathway through which social resources may relate to psychological wellbeing (Hu et al., 2022; Wu et al., 2024). Rather than acting solely through direct associations, perceived support may contribute to wellbeing by being associated with individuals’ adaptive coping capacities and psychological flexibility over time.

From a practical standpoint, the observed associations highlight the potential relevance of integrating resilience-oriented strategies into support-focused initiatives. Programs incorporating components such as mindfulness practices, cognitive-behavioral techniques, and stress management training may help strengthen individuals’ perceived ability to cope with challenges, thereby reinforcing the broader benefits of support systems. Framing such approaches within developmental contexts may enhance their sustainability and applicability across various stages of emerging adulthood.

### 4.3 The moderating role of gender (hypothesis 3)

Multi-group SEM analyses indicated that gender was a significant moderator of the associations among perceived social support, psychological resilience, and life satisfaction. Specifically, the associations between social support and resilience, and between resilience and life satisfaction, appeared stronger among women than among men.

These gender-related patterns may be interpreted within the sociocultural context of Turkey, which is shaped by collectivist values and traditional gender roles. Cultural expectations surrounding emotional expression, help-seeking behaviors, and caregiving responsibilities may influence how individuals engage with and appraise social support. Such gendered behavioral norms could help explain the differential associations observed across groups.

In Turkey, women are often more likely to engage in help-seeking behaviors and report emotional openness, potentially due to social norms that emphasize interdependence and relational connectedness (Çebi and Demir, 2020; Pejičić, 2024). Traditional gender roles that align with collectivist ideals—such as family cohesion and nurturance—may correspond more closely with women’s social experiences, which could be related to stronger associations between perceived support and wellbeing (Kaplan and Kaçkın, 2024; Bedrov and Gable, 2022).

Conversely, men may be more likely to encounter social norms that emphasize emotional restraint, self-reliance, and concern for family honor, which may be linked to lower levels of help-seeking (Kantar and Yalçın, 2023; Keskin and Karaman, 2020). For some men, internalized beliefs about masculinity could result in lower identification with psychological support, potentially corresponding to weaker observed associations between support-related constructs and wellbeing outcomes.

Socioeconomic factors may also contribute to these gendered dynamics. Higher levels of education, employment, and financial independence are often associated with greater engagement in help-seeking, particularly among women (Bayrakçeken et al., 2023; Güney et al., 2024). In contrast, men with lower socioeconomic status may encounter more stigma and practical barriers to accessing psychological support (Karaaslan et al., 2024). In addition, cultural norms related to emotional suppression and honor may be negatively associated with resilience, especially when psychological needs remain unexpressed (Günsoy et al., 2020).

These interpretations are consistent with prior research suggesting that women tend to show stronger associations with emotional and social support, possibly due to their reliance on expressive and relational coping styles (Zhang et al., 2014; Taylor et al., 2000). For example, Prowse et al. (2021) reported that female university students experienced higher levels of stress, loneliness, and mental health challenges during the COVID-19 pandemic compared to male students. Females were also more likely to use social media as a coping strategy, which was associated with increased stress and academic difficulties. These findings point to potential gender differences in coping mechanisms that may help contextualize the current study’s results.

Taken together, these culturally and socioeconomically informed gender-related patterns underscore the value of incorporating gender considerations into support-focused programming. Initiatives tailored for women may be more relevant when they emphasize emotional expression and relational engagement, whereas programs designed for men may benefit from integrating more structured, goal-directed, and autonomy-supportive elements that align with prevailing masculine norms.

### 4.4 Theoretical and practical implications

The findings of this study offer meaningful theoretical contributions to the literature on social support, psychological resilience, and life satisfaction. By employing a longitudinal design, the research provides evidence for a directional association between social support and life satisfaction, addressing limitations of prior cross-sectional studies (Lucas, 2007; Zhang, 2020). The results are consistent with the stress-buffering model (Cohen and Wills, 1985), indicating that social support may function as a protective factor against stressors, which is associated with greater long-term wellbeing. Furthermore, the mediating role of psychological resilience aligns with resilience theory (Luthar and Cicchetti, 2001; Masten, 2013), suggesting that resilience may be one of the mechanisms through which social support is linked to enhanced life satisfaction.

Building on these theoretical insights, the practical implications of these findings are equally noteworthy. Given that social support exerts a lasting positive influence on life satisfaction, mental health professionals, educators, and policymakers should focus on strengthening social support networks. Structured peer support programs in universities, workplace mentoring initiatives, and digital mental health platforms could enhance resilience and long-term psychological wellbeing. Moreover, resilience-building interventions such as mindfulness training, cognitive-behavioral strategies, and social skills development programs can further amplify the positive effects of social support.

In addition to these broad recommendations, the study highlights the importance of gender-specific interventions. The findings indicate that women benefit more from social support in enhancing resilience and life satisfaction, whereas men may require different approaches. Thus, interventions should be tailored accordingly:

For women: Programs should focus on expanding social support networks, particularly in professional and academic settings where stress levels are high. Group-based interventions, mentorship programs, and expressive coping strategies may be particularly beneficial.

For men: Since men may be less likely to seek emotional support, interventions should incorporate problem-solving training, stress management strategies, and structured resilience exercises. Leadership-based support groups could encourage men to utilize available social resources more effectively.

Furthermore, interventions should be age- and context-sensitive. While young adults in university settings may benefit from peer support initiatives, working professionals and older adults may require community-based programs tailored to workplace stress or family responsibilities. By recognizing these demographic variations, mental health initiatives can be more effectively tailored to meet the diverse needs of different populations.

### 4.5 Limitations

This study is not without limitations. The attrition rate of 27.5% over 1 year, while statistically accounted for, may still limit the generalizability of findings. Additionally, the reliance on self-report measures introduces potential biases, which future studies could address through multi-method approaches.

Cultural specificity is another limitation. Conducted within a Turkish context, the findings may not generalize to other cultural settings. Future research should examine the same model across diverse populations to assess the role of cultural norms.

Moreover, the unidimensional measurement of social support limits the granularity of insights. Future studies should differentiate between emotional, instrumental, and informational support to explore their unique contributions.

Finally, although the Cross-Lagged Panel Model (CLPM) used in this study enabled the examination of directional associations across time, it does not disaggregate within-person variability from between-person stability. This limitation may limit the interpretation of dynamic psychological processes that evolve over time. Recent methodological advances, such as the Random Intercept Cross-Lagged Panel Model (RI-CLPM), address this issue by allowing for the separation of intra-individual and inter-individual variance. Although CLPM was appropriate given the sample size and complexity constraints of the present study, future research would benefit from employing RI-CLPM or similar models to gain a more nuanced understanding of reciprocal associations.

### 4.6 Future research directions

Future studies should explore longer-term trajectories using extended longitudinal designs. Experimental approaches could strengthen causal inferences and help identify the active components of interventions.

Cross-cultural comparisons would illuminate the universality or context-dependence of these associations. Incorporating variables such as socioeconomic status, personality traits, and coping styles would enrich the understanding of individual differences.

Technology-based support systems, including digital peer support and mental health apps, also represent a promising avenue for future research, particularly in reaching underserved populations.

In addition to differentiating dimensions of social support, future studies could benefit from employing more advanced longitudinal modeling strategies. Methodologies that account for both within-person variability and stable individual differences may provide a deeper understanding of how social support and life satisfaction interact over time. Using such models could help clarify reciprocal dynamics and generate culturally responsive insights, particularly within non-Western contexts.

## 5 Conclusion

This study offers longitudinal evidence of associations between perceived social support and life satisfaction, both directly and through psychological resilience, with gender moderating these associations. These findings are consistent with theoretical perspectives that emphasize the role of social support in psychological wellbeing and may inform the development of context-sensitive support frameworks.

By taking into account social, psychological, and demographic factors, future programs may be better positioned to address the diverse needs of emerging adults and to promote sustained levels of wellbeing over time.

## Data Availability

The raw data supporting the conclusions of this article will be made available by the authors, without undue reservation.
